# Improving Real-Time Lower Limb Motor Imagery Detection Using tDCS and an Exoskeleton

**DOI:** 10.3389/fnins.2018.00757

**Published:** 2018-10-23

**Authors:** Marisol Rodríguez-Ugarte, Eduardo Iáñez, Mario Ortiz, Jose M. Azorín

**Affiliations:** Brain-Machine Interface Systems Lab, Systems Engineering and Automation Department, Miguel Hernández University of Elche, Elche, Spain

**Keywords:** transcranial direct current stimulation (tDCS), real-time, brain-machine interface (BMI), lower limb, exoskeleton, motor imagery (MI)

## Abstract

The aim of this work was to test if a novel transcranial direct current stimulation (tDCS) montage boosts the accuracy of lower limb motor imagery (MI) detection by using a real-time brain-machine interface (BMI) based on electroencephalographic (EEG) signals. The tDCS montage designed was composed of two anodes and one cathode: one anode over the right cerebrocerebellum, the other over the motor cortex in Cz, and the cathode over FC2 (using the International 10–10 system). The BMI was designed to detect two MI states: relax and gait MI; and was based on finding the power at the frequency which attained the maximum power difference between the two mental states at each selected EEG electrode. Two different single-blind experiments were conducted, E1 and a pilot test E2. E1 was based on visual cues and feedback and E2 was based on auditory cues and a lower limb exoskeleton as feedback. Twelve subjects participated in E1, while four did so in E2. For both experiments, subjects were separated into two equally-sized groups: sham and active tDCS. The active tDCS group achieved 12.6 and 8.2% higher detection accuracy than the sham group in E1 and E2, respectively, reaching 65 and 81.6% mean detection accuracy in each experiment. The limited results suggest that the exoskeleton (E2) enhanced the detection of the MI tasks with respect to the visual feedback (E1), increasing the accuracy obtained in 16.7 and 21.2% for the active tDCS and sham groups, respectively. Thus, the small pilot study E2 indicates that using an exoskeleton in real-time has the potential of improving the rehabilitation process of cerebrovascular accident (CVA) patients, but larger studies are needed in order to further confirm this claim.

## 1. Introduction

Transcranial direct current stimulation (tDCS) is a non-invasive brain stimulation technique based on weak direct electrical current transferred between electrodes (from anode to cathode) over the scalp in order to modulate the neural membrane resting potential (Nelson et al., 2014; Rodríguez-Ugarte et al., 2016b; Lefaucheur et al., 2017). It modifies cortical excitability in a polarity-specific manner (Coffman et al., 2014). This means that neural excitability is generated under the area of the anode because the current flow goes into the brain, whereas in the underlying cortex where the cathode is, inhibition of neural activity is produced because the current flow goes out from the brain (Filmer et al., 2014; Wiethoff et al., 2014). Furthermore, the use of this technique implies adjusting four parameters: current density, stimulation duration, electrode size and electrode position. The vast majority of the studies focus their tDCS experiments on improving the performance of the upper limbs, the speech, or the balance; where the areas stimulated are either the motor cortex, the frontal area or the cerebellum (Monti et al., 2013; Hortal et al., 2015; Foerster et al., 2017). In these studies, the range of current density used is typically between 0.04 and 0.06 mA/cm^2^ with a duration of 15 or 20 min (Marquez et al., 2013) and electrode sizes of about 35 cm^2^. However, there are just few studies that center their goals in meliorating lower limb performance and therefore, much remains to be investigated. In addition, stimulation with such big electrode surface areas gives only a vague idea of the areas of the brain that are important in producing the results.

Brain machine interfaces (BMIs) are a non-invasive technique that records and decodes electroencephalographic (EEG) signals to control an external device (Barrios et al., 2017). Two of the most common EEG-based BMIs are motor imagery (MI) and motor execution (ME). MI is defined as a mentally repetitive action without any overt motor movement (Park et al., 2013). Various functional magnetic resonance imaging (fMRI) studies have demonstrated that MI and ME activate common neural networks including the primary motor cortex (M1), supplementary motor area (SMA), premotor area (PM) and cerebellum (Allali et al., 2013; Hétu et al., 2013; Sharma and Baron, 2013; Zapparoli et al., 2013). Furthermore, MI is characterized by the decrease of power in the bands θ high (6–7 Hz), μ (8–12 Hz), and β (13–35 Hz) (Reynolds et al., 2015).

The purpose of this work is to test if a novel tDCS montage boosts the accuracy of lower limb MI detection using a real-time BMI. The tDCS montage is composed by three small electrodes that focus on the lower limbs: two anodes and one cathode. One anode is located over the right cerebrocerebellum, the other one over M1 in Cz, and the cathode over FC2 (using the International 10–10 system). Many studies have researched the stimulation just over the motor cortex or the cerebellum (Boehringer et al., 2013; Sehm et al., 2013; Clancy et al., 2014; Ferrucci and Priori, 2014), but never the two areas at the same time, like in this study. The effects of the stimulation over the cerebellum are still unclear, but recent studies showed an improvement of the task performed when the anode was over the cerebellum (Hardwick and Celnik, 2014; Bradnam et al., 2015). However, the anode over the cerebellum is also believed to cause neural inhibition over the motor cortex (Galea et al., 2009; Grimaldi et al., 2016). This is why a second anode was added over Cz. This anode supplied a slightly higher current than the one over the cerebellum to counteract this effect and to excite neural activity in M1.

Two single-blind studies, E1 and E2, were conducted where subjects were randomly separated into two groups: sham and active tDCS. The sham group received a fake stimulation while the active tDCS group was given 0.3 mA over Cz and 0.2 mA over the right cerebrocerebellum. A BMI based on power difference in θ, μ and β bands was designed to detect two MI tasks: relax and gait MI. Both experiments had a duration of five consecutive days (for each subject). The first one, E1, was based on visual cues and feedback. The second one, E2, was a smaller pilot test which was based on auditory cues, where subjects wore a lower limb exoskeleton as feedback. It should be noted that the combination of a real-time BMI with a lower limb exoskeleton and tDCS is quite challenging and has the strong potential of improving (via tDCS) the quality of many clinical applications that involve the real-time control of these machines. Indeed, the intention of this second setup is the later use on real-time rehabilitation therapies of cerebrovascular accident (CVA) patients with lesions on the right leg. The main output to measure the effectiveness of the experiments was the MI detection accuracy, but given the experiments' duration, the development of brain plasticity over the course of the 5 days was also analyzed. Our hypothesis was that the active tDCS group would obtain better detection accuracy results than the sham group.

## 2. Materials and methods

This work studies a novel tDCS montage with two different experimental setups regarding cues and feedback. The first one, called in this paper E1, gives visual cues and visual feedback, while the second, named E2, gives auditory cues with the feedback coming from the movement/non-movement of an exoskeleton. E2 is a smaller pilot test to check if the feedback of the exoskeleton provides an improvement of the results, so that it can possibly be used later in the rehabilitation of CVA patients.

### 2.1. Subjects

Twelve healthy subjects with a mean age of 26.9 ± 5.8 years old (age range 20–39) volunteered to perform E1 and four volunteers with a mean age of 25.8 ± 0.7 years old (age range 22–34) participated in E2. All of them received information prior to the experiment and gave written informed consent according to the Helsinki declaration. None of the subjects had a history of neurological and/or psychiatric diseases or was receiving medication during the experiment that could alter the central nervous system. The Ethics Committee of the Office for Project Evaluations (Oficina Evaluadora de Proyectos: OEP) of the Miguel Hernández University of Elche (Spain) approved the study.

### 2.2. Experimental design

The aim of both single-blind experiments was to detect two different cognitive states: relax and gait MI, using a real-time BMI based on EEG signals. For both experiments, initially subjects were randomly separated into sham or active tDCS groups of the same size (six participants in each group of E1 and two participants in each group of E2). For five consecutive days (Monday to Friday), each participant was subjected to one experimental session, which initiated with a period of stimulation. The sham group received 15 min of fake stimulation, while the active tDCS group received 15 min of real stimulation (more details in section 2.3).

#### 2.2.1. E1 experiment

**Figure 1:**
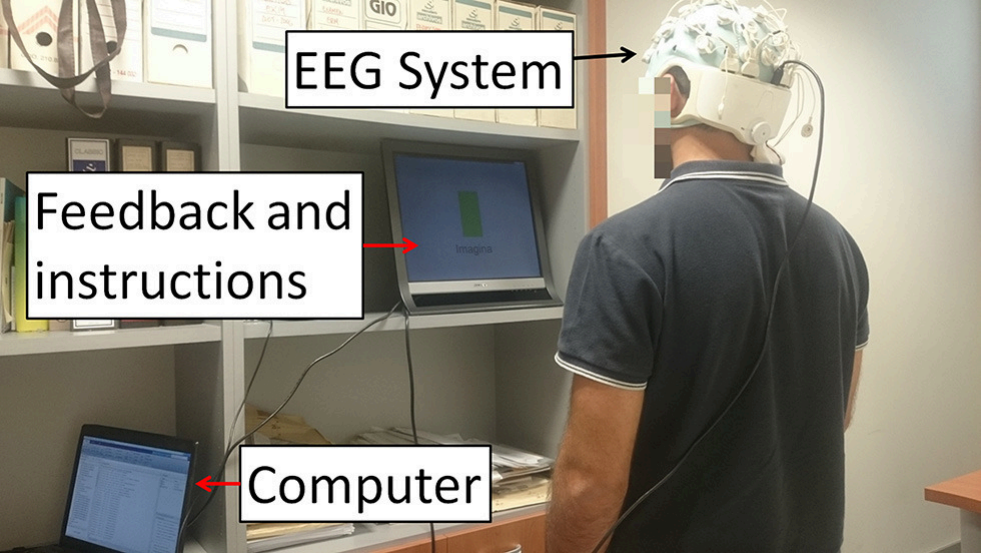
E1 experimental setup. Subjects stood in front of a screen that supplied instructions while their EEG signals were recorded. The instructions given were: *Relax, Imagine* and + (transition). During *Relax*, subjects had to clear their mind as much as possible. During *Imagine*, they had to visualize they were walking. Tasks appeared at random but two tasks of the same type never appeared more than twice in a row. The + (transition) period represented a transition to separate the *Relax* and *Imagine* tasks. Written informed consent was given by the subject to publish the photo.

**Figure 2:**
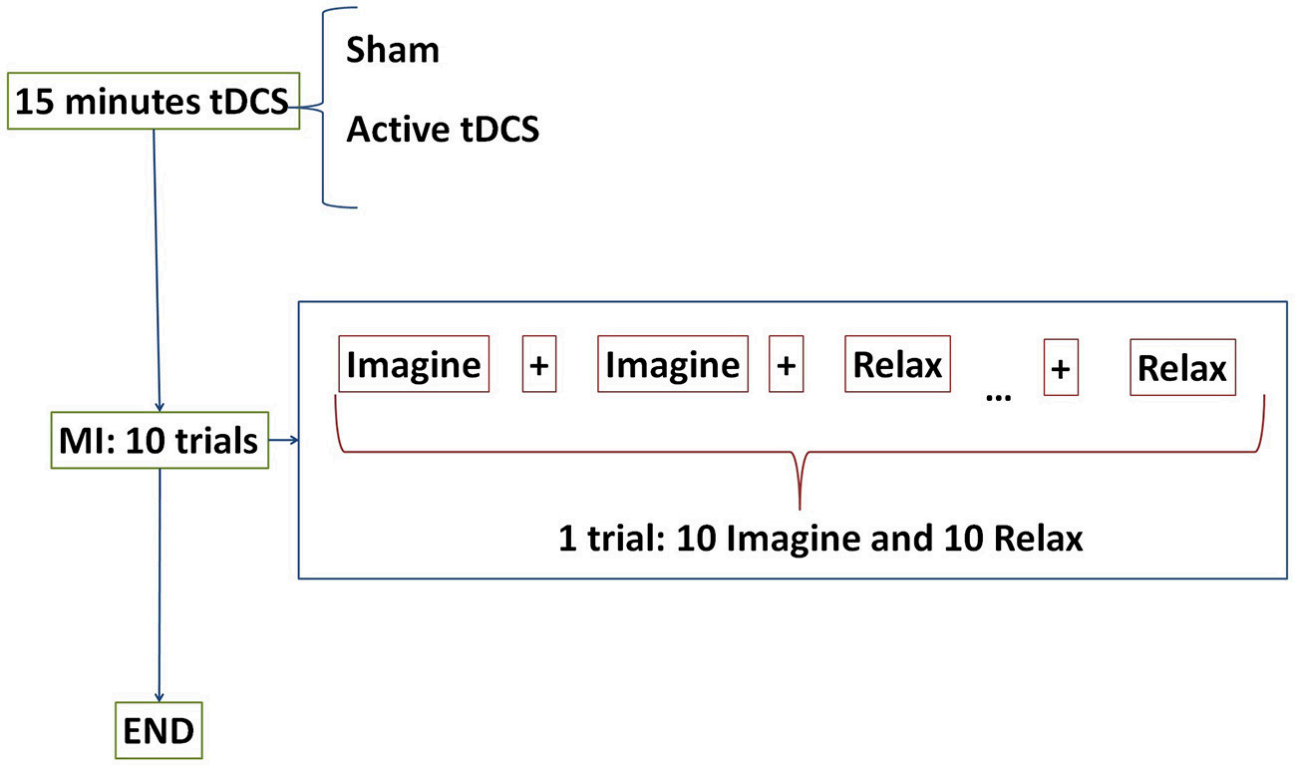
E1 temporal sequence on each day. Subjects were randomly separated into two groups: sham or active tDCS. During 15 min participants received the corresponding stimulation according to their group. After that, subjects performed 10 trials of motor imagery (MI) tasks. The tasks were composed of *Relax* and *Imagine* tasks separated by transition periods represented by + displayed on the screen. One trial consisted of 10 *Relax* and 10 *Imagine* tasks.

Participants performed one session each day for five consecutive days. One session was composed of the initial stimulation, followed by 10 MI trials. For each trial, subjects stood in front of a screen that provided instructions while their EEG signals were being recorded (Figure 1). Three types of instructions were supplied: *Relax, Imagine* and + (transition). During *Relax* periods, subjects had to clear their minds as much as possible; during *Imagine* periods, they had to imagine a gait movement. *Relax* and *Imagine* tasks appeared at random, but to avoid mind tiredness or getting bored, two tasks of the same type never appeared more than twice in a row. The transition periods, or + periods, separated different tasks of *Relax* or *Imagine*. *Relax* and *Imagine* lasted between 6 and 7.4 s, while the + (transition) periods lasted 3 s. Subjects were instructed to avoid blinking, swallowing, performing head movements or any other kind of artifact during the *Relax* and *Imagine* periods, postponing these actions to the + (transition) periods. Each trial consisted of 10 Relax and 10 Imagine periods. Figure 2 represents the temporal sequence of this experiment.

#### 2.2.2. E2 experiment

On the very first day, before any stimulation protocols, subjects were familiarized with the lower limb exoskeleton. They were mounted in the exoskeleton, and the exoskeleton was activated. Through verbal cues, the subjects were instructed to imagine gait until they felt comfortable that they were not trying to execute the motor task, but rather were imagining it. This pre-training phase was intended to remove any strong noise associated to the subjects trying to solely execute the movement later in the experiment.

**Figure 3:**
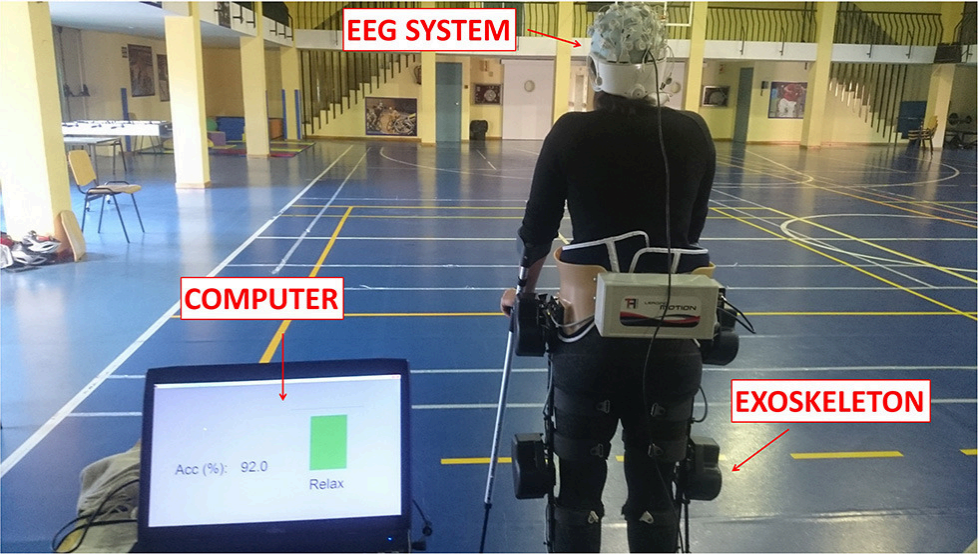
E2 experimental setup. Subjects stood wearing an exoskeleton while their EEG signals were recorded. Once the experiment started, subjects had to relax, clearing their mind as much as possible. Then, a beep auditory signal indicated to the subject to start gait imagery until they heard a double beep auditory signal. After this second beep, subjects had to relax again until the experimental trial finished.Written informed consent was given by the subject to publish the photo.

**Figure 4:**
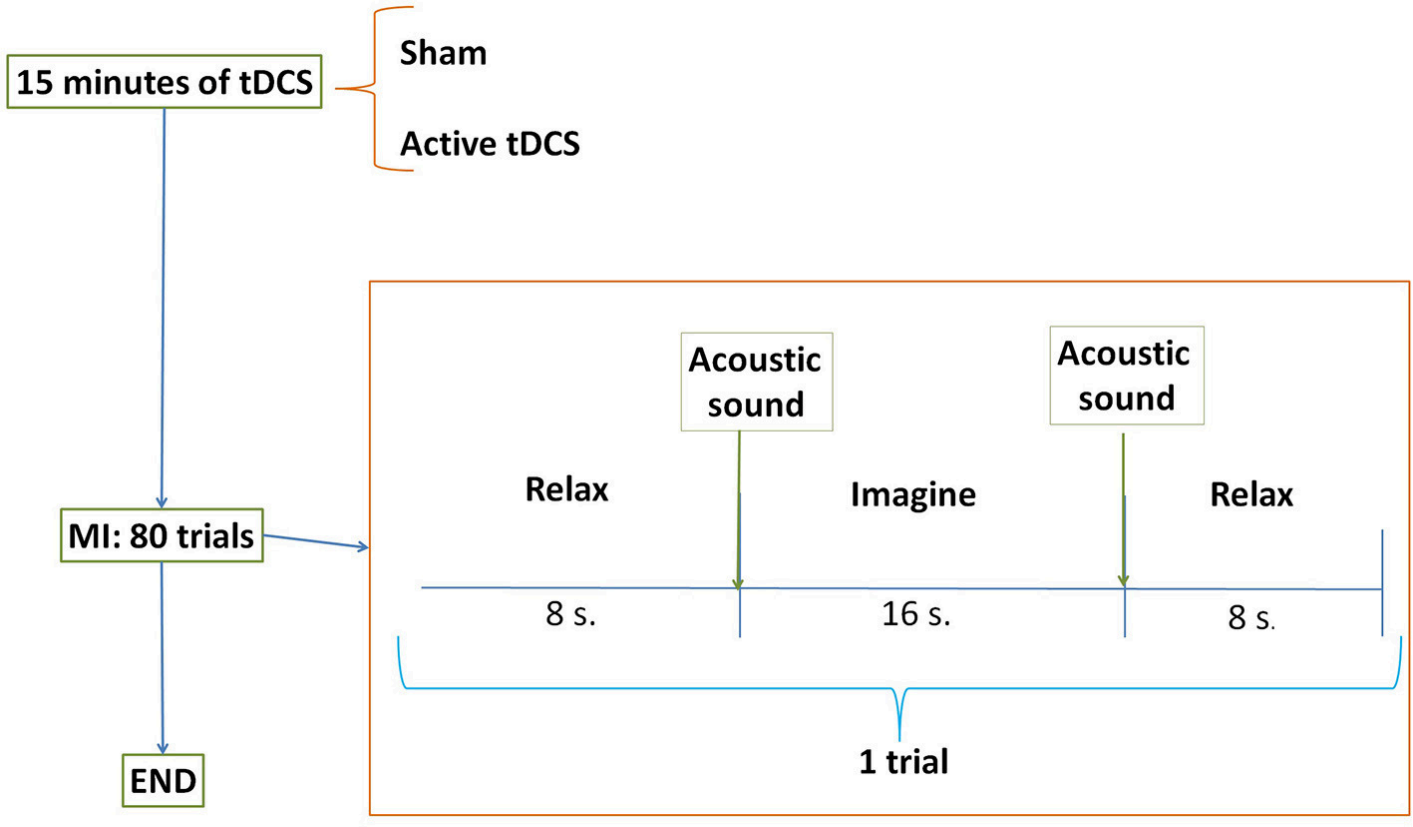
E2 temporal sequence on each day. Subjects were randomly separated into two groups: sham or active tDCS. During 15 min participants received the corresponding stimulation according to their group. After that, subjects performed 80 trials of motor imagery (MI) tasks. The trial was composed of two relax periods separated by one task of gait imagination.

Participants performed one session each day for five consecutive days. Throughout each session subjects stood wearing a lower limb exoskeleton while their EEG signals were recorded, as shown in Figure 3. One session was composed of the initial stimulation, followed by 80 MI trials. Each trial lasted around 35 s and was comprised of: an initial relax period where they had to clear their mind as much as possible; then, a beep auditory signal which indicated the subject to start the gait (walking) imagination until they heard a double beep auditory signal; after this, they had to relax again until the experiment finished. Therefore, there were two *Relax* periods which lasted 8 s each, separated by a longer *Imagine* period that lasted 16 s. A couple of seconds were needed to establish the connection between the BMI and the exoskeleton. Figure 4 represents the temporal sequence of this experiment.

In this experiment, the first 40 trials were used to train the BMI and the rest to test it. During the training, the exoskeleton moved by itself during the gait imagery period in order to provide the subjects with a more realistic feeling. Then, during the remaining 40 trials, the exoskeleton was turned off during the *Relax* periods and was activated according to the subject's EEG signals (i.e., using the BMI output) during the *Imagine* periods. The subjects were supposed to imagine the motor task instead of trying to execute it. More details on the BMI can be found in section 2.5.

### 2.3. Supply of tDCS

**Figure 5:**
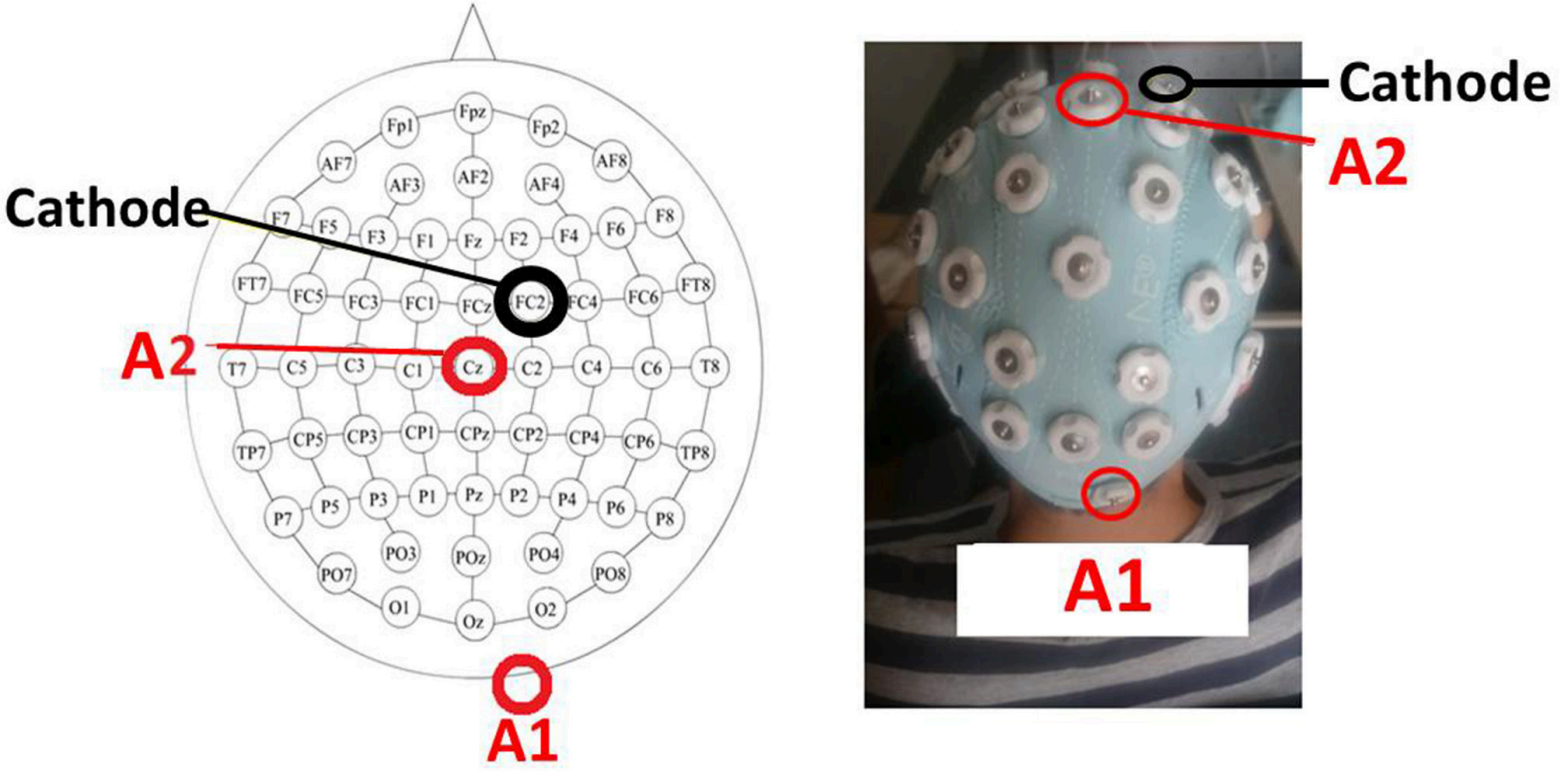
The tDCS montage. Placement of tDCS electrodes as a scheme **(Left)** and experimentally **(Right)**. The first anode (A1) is over the right cerebrocerebellum (two centimeters right and one centimeter down of the inion), the second anode (A2) is over Cz, and the cathode is over FC2.

As previously mentioned, the idea was to excite simultaneously the right cerebrocerebellum and the motor cortex because both areas are involved in motor imagery. To do that, one anode was located over the right cerebrocrebellum (two centimeters right and one centimeter down of the inion) and the other one over Cz on M1. The cathode was placed over FC2 (right hemisphere). Figure 5 shows a scheme of the position and placement of the electrodes. The cathode produces neural inhibition, meaning that the left hemisphere is being favored. This is because, in the future, the idea is to focus on patients that have suffered a CVA over the left hemisphere, which in turn affects their right lower limb.

The intensity was established to 0.2 and 0.3 mA for the cerebrocerebellum and Cz anodes, respectively. These intensities were chosen because anodal tDCS over the right cerebrocerebellum produces inhibition over the brain motor area (Angulo-Sherman et al., 2017), so to counteract this effect and excite the motor area, the second anode was placed over Cz with a slightly higher current. Using this configuration resulted in a cathode current density of 0.16 mA/cm^2^, which is higher than that used in most studies (about 0.06 mA/cm^2^). Having said that, this current density is well within the range of neurological safety that avoids brain damage (Bikson et al., 2016).

**Figure 6:**
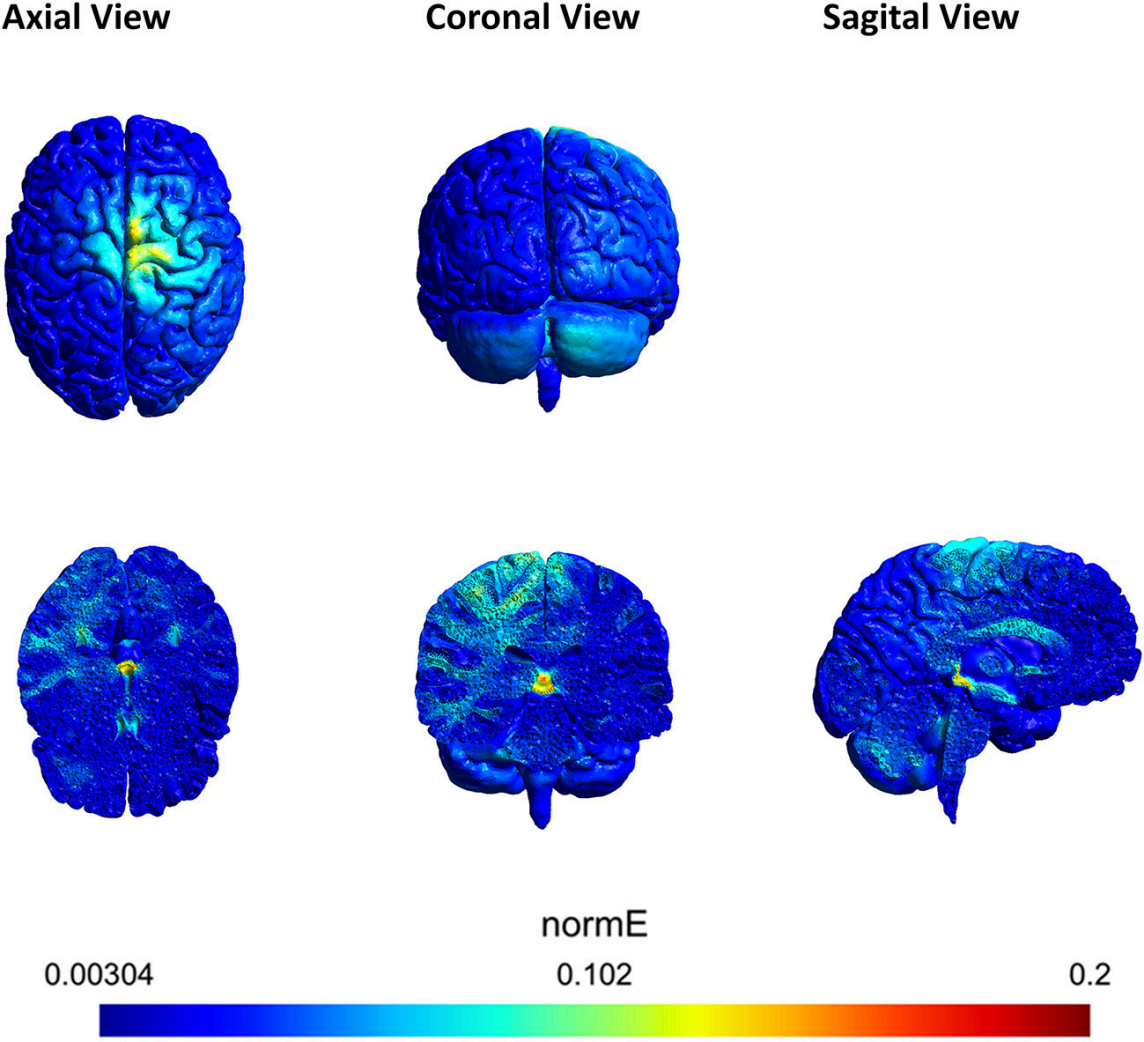
Axial, coronal and sagittal view of the tDCS simulation using SimNIBS. The scale represents the magnitude of the electric field (V/m) induced by the anodes A1 and A2. A1 was located over the right cerebrocerebellum and A2 over Cz. The cathode was located over FC2. A1 supplied 0.2 mA and A2 0.3 mA. The most affected area (red) is close to the red nucleus.

In order to corroborate that the areas of interest in the brain (motor area, right cerebrocerebellum, thalamus, contralateral hemisphere, red nucleus) were involved during the stimulation, an electric field simulation was carried out first. SimNIBS free platform (Thielscher et al., 2015) was used for the simulation. The parameters of the electrodes were set according to the materials employed in the experiments. All the electrodes were 1 cm of radius (surface area of π cm^2^), 3 mm of thickness and with 4 mm of space for the conductive gel. Figure 6 shows the magnitude of the electric field generated by the two anodes and one cathode in axial, coronal and sagittal views. The electric field produced was analyzed and it was confirmed that the sign of the electric field was negative over the cathode (showing directionality). Furthermore, the most affected area (red) is close to the thalamus and the red nucleus. Both areas belong to the cerebellum ascending output pathways to M1 and PM (Llinas and Negrello, 2015).

At the beginning of each experimental session, the StarStim R32 (Neuroelectrics, Barcelona, Spain) supplied direct current stimulation to the subject's brain. The duration was taken to be 15 min (each of the 5 days of the experiment), since various studies which treat different diseases obtained satisfactory results applying tDCS for that duration during 5 consecutive days (Marangolo et al., 2011; Bolognini et al., 2015; Ferrucci et al., 2016). Subjects in the active tDCS group were subjected to 15 min of such stimulation, while those in the sham group received a fake stimulation to create a placebo effect. This consisted of a 3 s ramp up followed by a 3 s ramp down to zero; then, 15 min of zero current; and lastly, another repetition of 3 s ramp up and ramp down.

### 2.4. EEG acquisition

The StarStim R32 (Neuroelectrics, Barcelona, Spain) was also used to acquire 30 EEG signals based on the International 10-10 system (P7, P4, CZ, PZ, P3, P8, O1, O2, C2, C4, F4, FP2, FZ, C3, F3, FP1, C1, OZ, PO4, FC6, FC2, AF4, CP6, CP2, CP1, CP5, FC1, FC5, AF3, PO3) with two reference electrodes (CMS and DRL) at a frequency of 500 Hz. The device was connected to the computer through a USB isolator.

### 2.5. Brain-machine interface (BMI)

Custom software in MATLAB (MathWorks Inc., Massachusetts, United States) was utilized for all data analysis. The first four trials of E1 and the first 40 trials of E2 were used to train a support vector machine (SVM) classifier with a radial basis function as kernel. This classifier was chosen because it was effective in previous studies and is one of the most robust classifiers (Rodríguez-Ugarte et al., 2016a). The SVM was in charge of categorizing data and determining if it belonged to relax or gait MI tasks. The remaining trials, six of E1 and 40 of E2, were utilized to test the BMI by measuring the detection accuracy, which was defined as the percentage of total correct classifications divided by the total number of classifications in each run.

Both training and test data in the two experiments were processed in very similar ways. The first 2 s of each task were discarded to assure the total concentration of the subject in the task and to get rid of the cue (visual or auditory) artifacts on the EEG. Data was processed in 1 s epochs each 0.2 s. For each epoch, the following process was carried out:

- a 4th order Butterworth high-pass filter with a cut-off frequency of 0.05 Hz was applied to remove the direct current;
- a Notch filter was used to eliminate the power line interference at 50 Hz;
- a 4th order Butterworth low-pass filter with cut-off frequency of 45 Hz was utilized;
- a Laplacian spacial filter was employed as in McFarland et al. (1997) to eliminate the influence of the other electrodes by means of weighting by their distance;
- nine electrodes from the M1, SMA and PM were selected: Cz, CP1, CP2, C1, C2, C3, C4, FC1, and FC2.

In both experiments, the training data was further analyzed. For each electrode, the power at each integer frequency from 6 to 35 Hz was calculated. This data was separated into relax and imagine groups for each frequency, and the frequency that attained the maximum power difference between relax and imagine was designated as the optimum frequency of that electrode. Finally, the power at the optimum frequency for each electrode was computed. Therefore, each epoch was associated with nine features (one for each electrode). Using the features, the SVM classifier was trained.

For the actual testing of the real-time BMI, the nine features of each epoch were computed using the power at the precomputed optimum frequencies from the training phase. Then the data was classified using the SVM classifier into relax or gait MI. As visual feedback, in E1 every correct classification resulted in the increase in size of a green bar shown in the screen. Meanwhile, in E2 the exoskeleton moved one step forward whenever three consecutive gait MI classifications were detected.

### 2.6. Exoskeleton

The lower limb robotic exoskeleton used was the H2 (Technaid, Madrid, Spain) designed by Bortole et al. (2015). The H2 has six degrees of freedom where hip, knee and ankle of each leg are powered joints. It was constructed for adults of heights between 1.5 m and 1.95 m and a maximum weight of 100 kg. The H2 has a lithium polymer battery of 22.5 VDC voltage and 12 Ah of capacity. It also has direct current (DC) motors to activate the joints actuators and sensors: potentiometers, Hall effect sensors, strain gauges and foot switches to determine the joint angles and human-orthosis interaction torques on the links.

The communication between the BMI and the H2 was through a bluetooth port. The connection was established in an Intel Core i7 laptop using MATLAB (MathWorks Inc., Massachusetts, United States) software. Each 0.5 s and during gait imagination periods, the BMI sent the user's output from the classifier to the exoskeleton.

### 2.7. Post-processing

#### 2.7.1. Statistical analysis

For the E1 experiment, data was analyzed via the Statistical Package Social Science (SPSS), version 22.0 (IBM Corporation, Armonk, NY, United States). The dependent variable was the classification accuracy and the independent variables were the group (sham or active tDCS) and the day of the experiment (from day 1 to day 5). Therefore, there were two types of studies: the difference between groups and the evolution of the performance of the subjects (here called plasticity) within groups. Hence, the appropriate statistical test to make was a mixed factorial ANOVA, but before doing so, the Kolmogorov-Smirnov (K-S) normality test was computed to check the existence of outliers. Then, for the study within groups, Mauchly's sphericity test was carried out to check the equality of the variances (Field, 2013). Lastly, the mixed factorial ANOVA analysis was completed. Furthermore, Bonferroni adjustments were applied for multiple pairwise comparisons between groups and within groups. A value of *p* < 0.05 was considered statistically significant.

For the E2 pilot experiment, the sample sizes were too small (two users per group) to rigorously justify the statistical analysis mentioned above. Therefore, the average accuracies were used directly to make the appropriate and relevant comparisons. Having said that, these results and their implications should come with a warning that this is only a preliminary study, and the sample sizes are small, so larger samples are needed to increase the accuracy of predictions.

#### 2.7.2. Analysis of optimal frequencies

As mentioned in section 2.5, based on the training data, an optimal frequency (where the greatest differences between relax and gait imagery was observed) was assigned to each electrode of each subject on any given day. These frequencies form a fundamental part of the model used to construct the BMI. Having said that, analyzing these frequencies independently provides more useful information. Indeed, after removing any outliers, it is possible to make a histogram of the optimal frequencies associated to each group on each day (each relevant subject in the group will have 9 optimal frequencies, one for each electrode, on any given day) that discriminates between three distinct frequency bands: high theta and mu rhythm (6–12 Hz), low and mid-range beta rhythm (13–20 Hz) and high beta rhythm (21–30 Hz). With this histogram, one can then determine the preferred frequency bands for each group and their evolution throughout the experiment.

#### 2.7.3. ERD/ERS analysis

Event-related desynchronization and synchronization (ERD/ERS) are EEG fluctuations during cognitive or motor processes. They are highly frequency-band specific and while ERD represents an increase of excitability, ERS represents the opposite (Pfurtscheller, 2001). For an electrode *e*, and for a fixed frequency *f*, let

(1) $$\begin{array}{l} ERD_{e}\left(f\right)=(\frac{G\left(f\right)-R\left(f\right)}{R\left(f\right)}) \end{array}$$

where *G*(*f*) is the average of the power at the frequency f over all gait-imagery-epochs, and *R*(*f*) is the same but averaged over all relax-imagery-epochs. Low values of *G*, resulting in negative values of *ERD*_*e*_, represent ERD, while higher values of *G*, resulting in positive values of *ERD*_*e*_, represent ERS. To obtain an average value of *ERD*_*e*_ over a frequency band, simply average over all integer frequencies, *f*, of interest (e.g., in the 6–12 Hz band, it would be the average of *ERD*_*e*_(*f*) for *f* = 6, 7, 8, 9, 10, 11, 12). A frequency-band *ERD*_*e*_ can be calculated for each electrode on each day of the experiment for each subject. This allows to produce a topographic map of the variable in the scalp, which one can then analyze to determine patterns of activation across the different areas of the brain.

## 3. Results

### 3.1. E1 experiment

The normality test indicated that there was an outlier within the sham group. This subject was removed from the data.

#### 3.1.1. Effects of tDCS in MI

**Figure 7:**
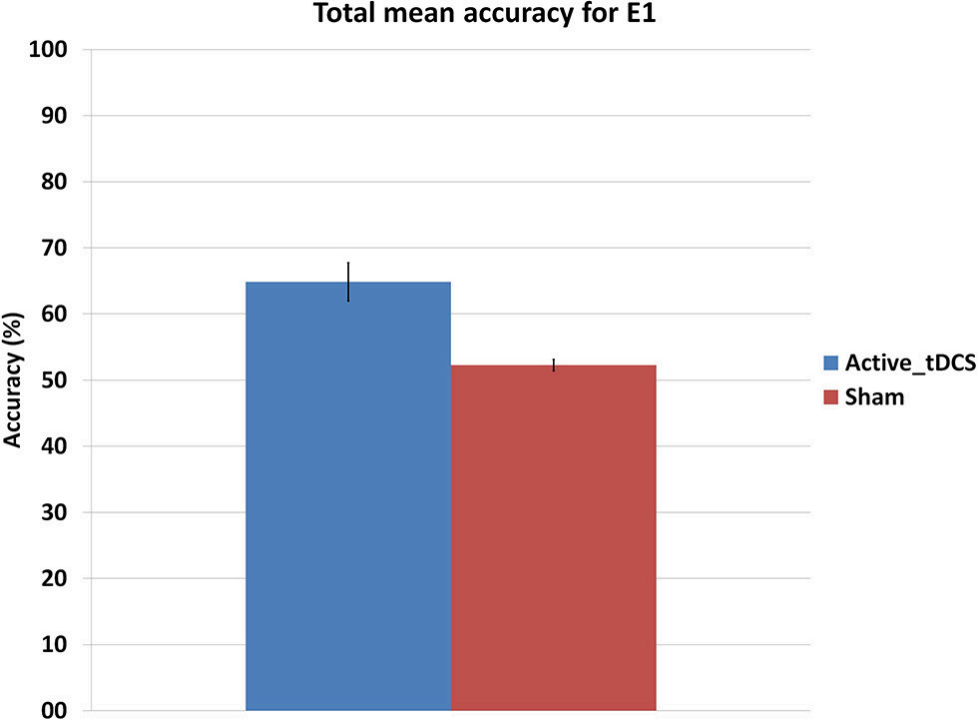
Mean accuracy for each group in the E1 experiment. The error bars indicate a standard deviation from the mean.

This section studies if there exist any effects of tDCS on the subjects. Results from the mixed factorial ANOVA showed that subjects were significantly affected by the group they belonged, *F*_(1, 9)_ = 9.47, *p* < 0.05. Figure 7 shows the mean accuracy achieved by each group, with the tDCS and sham groups getting 65 and 52.4% of detection accuracy, respectively.

**Figure 8:**
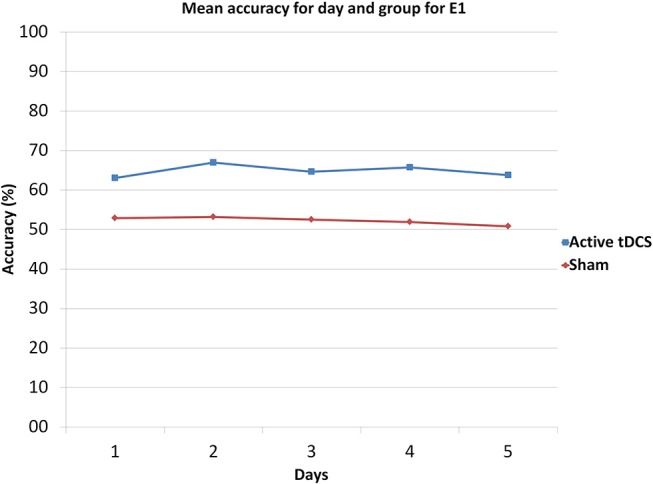
Mean accuracy for each day and group in E1.

Moreover, the comparison was broken down on a day by day basis, by making pairwise comparisons. Table 1 shows the *p*-values of those comparisons and Figure 8 illustrates the mean accuracy achieved by each group on each day. The results show that there were significant differences (*p* < 0.05) between the sham and tDCS groups from the second day onwards.

**Table 1:** Pairwise comparison of detection accuracy for each day between the tDCS and sham groups (E1).

**Day**	***p*-value**
1	0.06
2	0.04^*^
3	0.04^*^
4	0^*^
5	0.02^*^

The values statiscally significant were indicated with the ^*^ symbol.

#### 3.1.2. MI plasticity

This section analyzes the interaction effects between the days within groups. The results of Mauchly's test of sphericity show that the condition of sphericity was met, χ^2^(9) = 17.52, *p*>0.05, so it was not necessary to apply a correction factor.

The mixed factorial ANOVA showed no significant interaction between the days and the group, *F*_(4, 36)_ = 0.27, *r* = 0.1, *p*>0.05, meaning that there does not seem to be any major plasticity development throughout the 5 days of the experiment.

#### 3.1.3. Optimal frequencies and ERD/ERS results

A histogram showing the percentage of electrode optimal frequencies lying in the relevant frequency bands (high theta and mu rhythm, low and mid-range beta rhythm, and high beta rhythm) for each group and day of the E1 experiment is shown in Table 2. Clearly, the preferred frequency band is the high theta and mu rhythm (6-12 Hz).

**Table 2:** E1 histogram of optimal frequencies for each day and group.

**Group**	**Frequency range**	**Day 1 (%)**	**Day 2 (%)**	**Day 3 (%)**	**Day 4 (%)**	**Day 5 (%)**
Active tDCS	(6–12) Hz	81.5	77.8	81.5	85.2	79.6
	(13–20) Hz	11.1	13.0	11.1	7.4	16.7
	(21–30) Hz	7.4	9.3	7.4	7.4	3.7
Sham	(6–12) Hz	75.6	57.8	68.9	64.4	64.4
	(13–20) Hz	4.4	17.8	4.4	28.9	20.0
	(21–30) Hz	20.0	24.4	26.7	6.7	17.8

Since the high theta and mu rhythm (6–12 Hz) was the preferred frequency band, on each day of the E1 experiment and for each electrode, *e*, the variable *ERD*_*e*_ was averaged over all subjects common to a group (excluding outliers) and over the relevant frequency band (6–12 Hz). The resulting topographic map for the active tDCS and the sham groups is shown in **Figure 11** (top).

### 3.2. E2 experiment

#### 3.2.1. Effects of tDCS in MI

**Figure 9:**
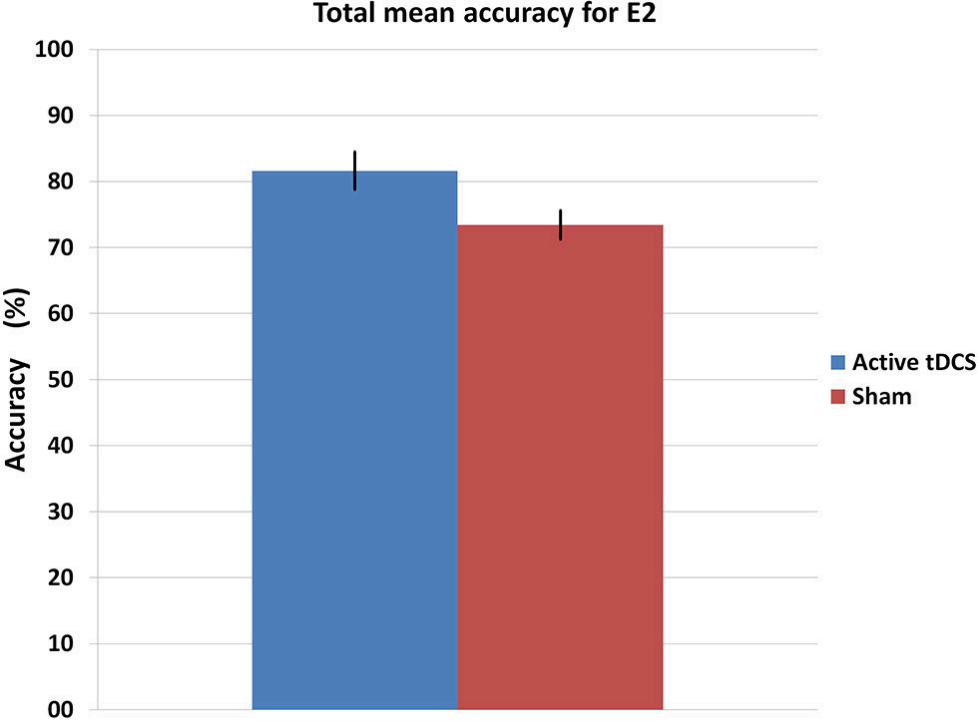
Mean accuracy for each group in the E2 experiment. The error bars indicate a standard deviation from the mean.

**Figure 10:**
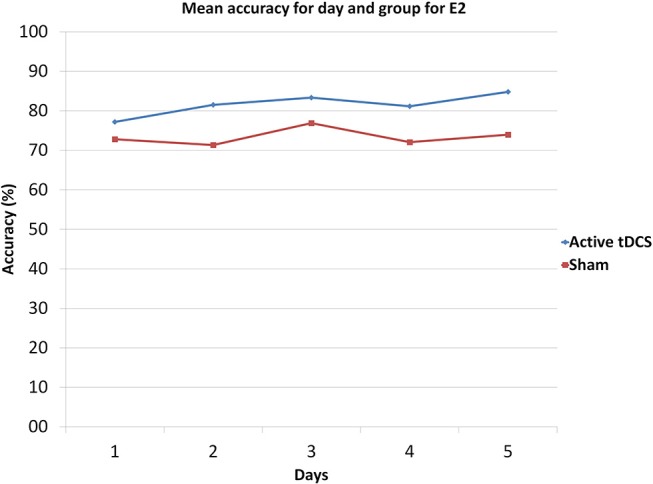
Mean accuracy for each day and group in E2.

Figure 9 shows the mean accuracy achieved by each group, with the tDCS and sham groups getting 81.6 and 73.4% of detection accuracy, respectively. Furthermore, Figure 10 illustrates the mean accuracy achieved by each group on each day, and there does not seem to be any significant changes in the accuracy as the days progress for either group (i.e., no plasticity is evident). Having said this, due to the preliminary nature of the E2 pilot study, these results have limitations as they involve very small sample sizes (two subjects per group), and larger data sets are necessary to be able to produce more robust results from the statistical standpoint.

#### 3.2.2. Optimal frequencies and ERD/ERS results

As in section 3.1.3, the associated histogram for E2 is shown in Table 3. The preferred frequency band was once again the high theta and mu rhythm (6–12 Hz).

**Table 3:** E2 histogram of optimal frequencies histogram for each day and group.

**Group**	**Frequency range**	**Day 1 (%)**	**Day 2 (%)**	**Day 3 (%)**	**Day 4 (%)**	**Day 5 (%)**
Active tDCS	(6–12) Hz	100.0	100.0	100.0	100.0	94.4
	(13–20) Hz	0.0	0.0	0.0	0.0	0.0
	(21–30) Hz	0.0	0.0	0.0	0.0	5.6
Sham	(6–12) Hz	66.7	66.7	50.0	50.0	50.0
	(13–20) Hz	0.0	0.0	0.0	0.0	0.0
	(21–30) Hz	33.3	33.3	50.0	50.0	50.0

**Figure 11:**
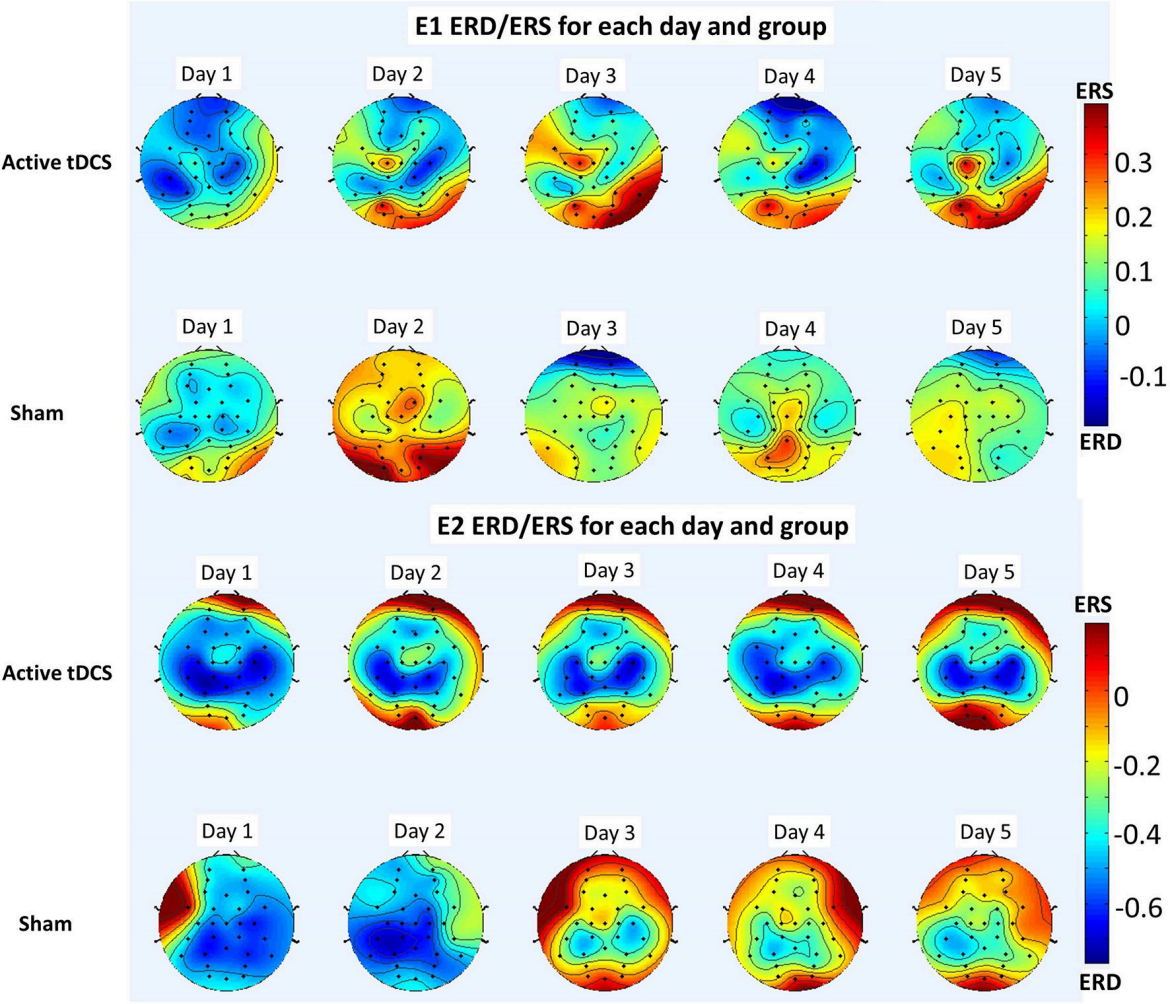
Topographic maps showing ERD (red) and ERS (blue) for the 6–12 Hz frequency band averaged over all participants for each day and group of each experiment. The results of E1 are shown in the top and those of E2 are shown in the bottom.

Meanwhile, the analogous topographic map for E2 for the preferred frequency band (6–12 Hz) is shown in Figure 11 (bottom).

## 4. Discussion

The results of E1 and the preliminary results of the pilot test in E2, seem to support the hypothesis that this novel tDCS montage improves the real-time classification of lower limb MI tasks. Before discussing the specific results further, a deeper neurological explanation for why the tDCS montage seems to have successfully worked is merited. The aim of the setup was to enhance the brain's learning abilities while stimulating the motor cortex which is responsible for lower limb movement (and imagination). With this in mind, an anode was placed over the cerebellum, since this improves the brain's learning abilities according to several studies (Mandolesi et al., 2003; Ferrucci et al., 2013; Shah et al., 2013; Hardwick and Celnik, 2014). However, placing this anode over the cerebellum also has other consequences. Namely, it produces the activation of Purkinje cells which inhibit the dentate nucleus and provoke disfacilitation of the motor cortex (Grimaldi et al., 2014; Cengiz and Boran, 2016; Lefaucheur et al., 2017), which is the opposite of what is desired regarding the activation of the motor cortex. For this reason, to counteract the effect of the first anode and excite the neural activity of the motor cortex, a second anode was placed directly in Cz over the motor cortex, and with a slightly higher current. Indeed, the currents used were 0.2 mA for the first anode and 0.3 mA for the second anode. The tDCS electrodes were not in direct contact with the skin, but rather with the hair. This reduced the probability of skin burns (Wang et al., 2015), which were not observed during the experiments (participants were encouraged to report any discomfort, but none was reported in association with the tDCS).

The active tDCS group achieved average detection accuracies of 65 and 81.6% for E1 and E2, respectively. When compared to the sham group, the active tDCS group obtained 12.6 and 8.2% higher accuracy performance in E1 and E2, respectively (Figures 7, 9). In addition, the active tDCS group of E1 was at least 10% better than the sham group at each given day (see Figure 8), while in E2, it was at least 4% better on each day (see Figure 10). Lastly, this data and the *p*-values from Table 1 indicate that from the second day onwards, the active tDCS group obtained significantly different and better results than the sham group in E1.

These conclusions are further supported with the results of analyzing the optimal frequencies and the ERD/ERS patterns in the brain. Regarding the optimal frequencies, Table 2 and the preliminary results of Table 3 show the stability of the frequency band trained, which in both cases corresponded to the high theta and mu rhythms (6–12 Hz). In E1 (Table 2) the preferred frequency band for the active tDCS group represented at least 78% of the optimal frequencies on any given day, while for the sham group it varied between 57 and 76%. The results of E2 show an even starker difference, with at least 94% of optimal frequencies lying in the preferred frequency band for the active tDCS group, while they ranged between 50 and 66% for the sham group. This seems to indicate that the tDCS favors a specific frequency band to train the new task.

Moreover, the ERD/ERS analysis shows that overall for both E1 and E2, there seems to be more desynchronization (ERD) on the mu rhythms of the tDCS group than in the sham group (see Figure 11). Furthermore, this mu wave desynchronization is occurring mostly in the sensorimotor area, as is reported widely in the literature when there is either motor execution or motor imagery (Pfurtscheller and Da Silva, 1999; Matsumoto et al., 2010). This desynchronization seems to be more evident in the preliminary study E2 than in E1, but in both cases it is observed. Thus, the active tDCS group for both experiments appears to enhance the modulation of the mu rhythm and the BMI control.

As observed from Figures 8, 10, for both experiments, the changes in accuracy for each group as the days progressed seems to have been minimal. Thus, one can say that there was little plasticity developed in the brain during the 5 days of the experiment. This is probably due to the simplicity of the task and the fact that the brain could have quickly adapted to this task early on in the training phase of the experiment of the first day.

Comparing the differences between E1 and E2 is very interesting but one must be careful in rushing to any conclusions, as the experimental protocols were different, and more importantly, the results of E2 are only preliminary at the time. Overall, E2 produced better accuracy results than E1: the active tDCS and sham groups of E2 were 16.7 and 21.2% more accurate than the respective groups of E1. Some differences in the protocol that could have led to these results, are that the duration of *Relax* and *Imagine* periods between the two experiments was different; and more notably, that the nature of the cues and feedback was different as well. Indeed, it should be mentioned that all subjects in E1 reported frustration about the visual feedback (a green bar that increased with each real-time correct detection), saying that they became anxious when the green bar did not move. Naturally, this could have affected the results. Meanwhile, in E2 the feedback was much more natural as it involved movement of the body. In fact, no such frustration was reported by the users in E2.

Comparing the results of E1 and the preliminary results of E2 through the ERD/ERS analysis is also of interest (see Figure 11). Indeed, the desynchronization is observed to be stronger and more consistent in E2 than in E1. This seems to be consistent with some results in the literature involving upper limb exoskeletons (Gomez-Rodriguez et al., 2011), which found the discriminative power of the sensorimotor area to be higher when using an exoskeleton, thus providing a benefit in terms of the resulting BMI designed.

It should be noted that the pilot test E2 was a challenging experiment as it involved combining tDCS with a real-time BMI connected to an exoskeleton. Exoskeletons are often simply pre-programmed or controlled directly through third party interfaces (joysticks, cellphone applications, etc.), but only until relatively recently have they begun to be controlled via BMIs. Designing a real-time BMI is also not trivial in itself (it is sometimes preceded by the design of offline BMIs). Thus, the study of real-time BMI control of exoskeletons is only starting and has many potential clinical applications, especially in the rehabilitation of patients. Thus, combining this concept with tDCS, which is aimed to improve and accelerate cognitive ability, enriches and increases those applications even more. Indeed, the intention is to use this setup in the future to enhance the recovery of CVA patients with an affected lower right limb. Having said that, the study carried out here was only a preliminary pilot study involving only a few subjects. To confirm the results, a larger sample of subjects or even patients is necessary, but the limited results obtained for now look promising.

Some final comments are warranted regarding the real-time functioning of the exoskeleton in E2. To have a realistic usability of the BMI with the exoskeleton, the analysis of the false detections during relax periods is important, and reducing it is an essential objective. The rate of such detections is referred to as the false positive rate, or FPR (which is the complement of the accuracy when restricted to only relax periods). When averaging both groups in E2, the FPR was 11.7% (equivalently, an accuracy of 88.3% during relax), with an FPR of 11.3% for the tDCS group and of 12.1% for the sham group. The values for both groups were very similar, which shows that the overall increase in accuracy resulting from the stimulation of the tDCS group, was due to an increase in accuracy during the imagination periods (indeed, the accuracy on those periods was 92.7% for the tDCS group and of 80.4% for the sham group). In any case, overall, these values of FPR seem reasonable for this preliminary experiment, but reducing them further should be a future design goal.

## 5. Conclusion

A novel tDCS configuration was successfully designed to improve the detection of two MI tasks (relax and gait MI) using a real-time BMI. Two anodes and one cathode were used: one anode was located over the right cerebrocerebellum and supplied 0.2 mA, the other anode was over Cz and supplied 0.3 mA, and the cathode was located over FC2. Two single-blind experiments, E1 and E2, were carried out, where subjects were randomly separated into two groups of the same size: sham and active tDCS. The sham group received a fake stimulation while the active tDCS group was truly stimulated. E1 involved twelve healthy subjects in total who received visual instructions and real-time feedback through a screen. Meanwhile, E2 was a pilot study involving only four healthy subjects who received auditory cues and wore a lower limb exoskeleton as feedback. E2 has potentially many clinical applications in the future. In particular, it can be used in the rehabilitation of patients that have suffered a cerebrovascular accident (CVA) affecting their right lower limb. The analysis indicated differences between the active tDCS and sham group in both experiments. The active tDCS group achieved 12.6 and 8.2% higher detection accuracy than the sham group in E1 and E2, respectively, reaching 65 and 81.6% mean accuracy in each experiment. Furthermore, the preliminary results indicate that the exoskeleton (in E2) enhanced the detection of the MI tasks with respect to the visual feedback (in E1), increasing the accuracy obtained in 16.7 and 21.2% for the active tDCS and sham groups, respectively. Having said that, more studies with larger samples of actual patients are needed to validate this observation.

## Author contributions

MR-U is responsible for the design, implementation, acquisition and data analysis. In addition, EI and MO supervised the work and contributed with the revision process. JA actively contributed as director of the work.

### Conflict of interest statement

The authors declare that the research was conducted in the absence of any commercial or financial relationships that could be construed as a potential conflict of interest.

## Abbreviations

tDCS: transcranial direct current stimulation
MI: motor imagery
ME: motor execution
BMI: brain-machine interface
EEG: electroencephalographic
fMRI: functional magnetic resonance imaging
M1: primary motor cortex
SMA: supplementary motor area
PM: premotor area
CVA: cerebrovascular accident
SVM: support vector machine.
